# News Items

**Published:** 1919-02

**Authors:** 


### NEWS ITEMS.

It is authoritatively stated that the epidemic of influenza in South Africa has resulted in a financial loss to the leading insurance companies of approximately $7,500,000. One insurance manager said it was a startling fact that in the course of a few weeks the epidemic had cost the companies more than they had been called upon to pay for all of their war risks.

---

Satisfaction is expressed by the Times (London) in an editorial commenting upon the foundations of an ''inter-Allied fellowship of medicine,'' which it says, will unite more closely American, British and other allied schools of medicine. Dr. Sir William Osler is president of the organization.

---

Threatened with extinction by influenza, the population of the island of Tahiti, a French possession in the Society Islands group in the Pacific, still is awaiting word of the sending of relief in response to repeated and urgent wireless appeals sent since early in December. When the first cablegrams asking aid came the American Red Cross, besieged by appeals from nearly every quarter of the globe, decided that nothing could be done for the distant islanders. They hoped the epidemic would wear itself out in a few weeks, or long before a relief ship could make the voyage. In the past few days news dispatches have announced that the scourge has run its course, but nevertheless belated plans for aid are going forward while an answer to an official query is awaited. More than one-seventh of Tahiti's population of about 12,000 inhabitants was dead a month ago and the older generations of natives practically was wiped out.

---

Dr. C. W. Goddard of Holland, Bell County, whose appointment as State Health Officer and president of the State Board of Health by Governor Hobby was recently confirmed by the Senate, has qualified and taken charge of the health department. Dr. Goddard succeeds Dr. W. B. Collins, who served as State Health Officer four years.

---

Postponement of ''Health Sunday'' from February 9 to February 23, so as not to conflict with the Theodore Roosevelt memorial services arranged for the earlier date, has been announced by Surgeon General Blue of the United States Public Health Service.

---

The recent influenza epidemic in South Africa caused nearly 129,000 deaths, including 1,176 whites, according to a statement by the minister of the interior.

---

The influenza epidemic has occasioned a known loss to insurance companies of $52,000,000 for claims made between October 1 and December 24.

---

The virus of trench fever and that of influenza and of some forms of nephritis have been isolated and identified, according to a report submitted to the director general of the army medical service in France by a number of army medical officers who have been investigating the causes of these diseases. According to this official statement, the virus in each case has been proved to be a minute globular cell varying in size and behavior in three types of disease. Investigations which have been conducted have resulted, it is believed, in the isolation of the germs of mumps, measles and typhus, the causes of which have been obscure and the bacilli of which have never before been isolated.

---

A forged prescription for narcotic drugs bearing the signature of a doctor who has been dead for several weeks, proved the undoing of an alleged drug addict in Dallas. When the prescription, dated Feb. 4, was presented at a downtown store the police were immediately notified and arrested the man. A hypodermic needle was found in his pocket.

